# Computational Analysis of Enhanced Magnetic Bioseparation in Microfluidic Systems with Flow-Invasive Magnetic Elements

**DOI:** 10.1038/srep05299

**Published:** 2014-06-16

**Authors:** S. A. Khashan, A. Alazzam, E. P. Furlani

**Affiliations:** 1 Department of Mechanical Engineering, United Arab Emirates University, 15551, Al-Ain, UAE; 2 Department of Mechanical Engineering, Khalifa University, Abu Dhabi, UAE; 3 Department of Chemical and Biological Engineering, University at Buffalo, NY, USA; 4 Department of Electrical Engineering, University at Buffalo, NY, USA

## Abstract

A microfluidic design is proposed for realizing greatly enhanced separation of magnetically-labeled bioparticles using integrated soft-magnetic elements. The elements are fixed and intersect the carrier fluid (flow-invasive) with their length transverse to the flow. They are magnetized using a bias field to produce a particle capture force. Multiple stair-step elements are used to provide efficient capture throughout the entire flow channel. This is in contrast to conventional systems wherein the elements are integrated into the walls of the channel, which restricts efficient capture to limited regions of the channel due to the short range nature of the magnetic force. This severely limits the channel size and hence throughput. Flow-invasive elements overcome this limitation and enable microfluidic bioseparation systems with superior scalability. This enhanced functionality is quantified for the first time using a computational model that accounts for the dominant mechanisms of particle transport including fully-coupled particle-fluid momentum transfer.

The ability to selectively separate biomaterial such as cells, proteins, genes and pathogens from a specimen for detection and characterization is fundamental to fields such as microbiology, medical diagnostics and biochemical sensing. The use of microfluidic devices for bioseparation has grown rapidly over the years due to advances in device technology. Microfluidic systems offer advantages over conventional batch separation systems. They enable efficient and rapid analysis of small samples with high reliability and low cost as well as the integration of multiple process steps in a single portable device format such as found in Lab-on-a-chip and micro total analysis systems (μTAS)^1^,^2^. Among the many methods used for bioseparation, magnetic-based systems have features that are especially attractive for many applications^3^,^4^,^5^,^6^,^7^. In these systems, the target biomaterial is first labeled with functionalized magnetic particles^8^,^9^,^10^. The labeled biomaterial is then separated as it flows through a microchannel using integrated magnetic micro-elements. These can be magnetically passive such as nickel-based microbars^11^,^12^,^13^,^14^,^15^,^16^,^17^,^18^ or active voltage-driven conductors^19^,^20^,^21^,^22^,^23^,^24^. In either case, the magnetic elements generate a localized high-gradient field that produces a force to sort or immobilize labeled biomaterial under flow. Passive soft-magnetic elements, as considered here, are magnetized using an external field and they return to an unmagnetized state once the field is removed. Thus, the force they produce can be switched on and off by applying and removing a bias field, thereby enabling the separation of a target biomaterial from a sample and its subsequent release in higher concentration on demand for further analysis. The relatively low magnetic susceptibility of the aqueous carrier fluid and unlabeled biomaterial provides good contrast between labeled and unlabeled material, which enables a high degree of selectivity.

In this paper a microfluidic design is proposed for separating magnetic or magnetically-labeled particles in a microfluidic system using integrated flow-invasive soft-magnetic elements. The elements are anchored in the system and are immersed in the carrier fluid (i.e. flow-invasive) with their length oriented transverse to the flow. This is in distinct contrast to more conventional systems wherein the elements are integrated outside of the flow channel, i.e. in the base or side walls of the channel. The particle capture efficiency in the conventional systems greatly diminishes as the size of the flow channel increases because of the relatively short range effectiveness of the magnetic force. Various magnetic configurations have been proposed to overcome this limitation. Sinha et al. (2009)^25^ used an electromagnet consisting of an enamel-coated wire wound around a tapered iron core to generate a large magnetic field gradient at its tip. Wie et al. (2014)^26^ proposed the use of micro rhombic magnets, each having a standing length of 40 µm along the long axis and a maximum thickness of 5 (or 8) µm along the minor axis. These elements, located in proximity to both sidewalls of the channel, become uniformly magnetized along the long axis due to the large magnetic anisotropy. Xia et al. (2006)^27^ utilized a layer of soft-magnetic material (NiFe) with a microcomb configuration that has a triangular saw-tooth edge positioned close to the side of the channel. They reported that the separation performance of this layer can be improved by setting its thickness equal to the height of the channel even at high volume throughput. Lin et al.(2007)^28^ exploited a side channel, located adjacent to the main flow channel, to inject small nickel particles to serve as magnetic field concentrators. They reported that the nickel particles improved the magnetic field gradient over a distance of 20 µm into the main flow channel. For enhancing the magnetic flux density gradient, Kang et al. (2008)^29^ electroplated a nickel bar alongside the edge corner of the main separation channel. Ramadan et al. (2006)^30^ utilized a multi-layer MEMS fabrication technology to integrate micro-coils and magnetic pillars in order to generate a magnetic field gradient. Deng et al. (2002)^31^ used soft lithography and electro deposition techniques to fabricate an array of short nickel posts (7 µm in height and 15 µm in diameter) to serve as magnetic elements in a microfiltration device. Once magnetized, the posts generated strong magnetic field gradients and trapped superparamagnetic beads in a flowing stream of water. Their study did not report on the overall capture efficiency but considering the very low aspect ratio of these posts, it is expected that the active magnetic capture region is limited to tens of microns from the posts. Other less flow-invasive continuous flow designs incorporated microfabricated ferromagnetic strips (MFS) onto the floor of the microchannel to generate the required magnetic field gradients and therefore direct the beads (cells) into target outlets^32^,^33^, in accordance with their direction. Han and Frazier (2004)^34^ constructed externally flow-invasive magnetizable wires into the channel structure that spanned the entire height of the channel for directing blood cell separation into target outlets. As a remedy for the short-ranged magnetic force, the interior design of the channel can be re-structured to force the labeled biomaterial closer to the magnetic elements. This can be achieved using microfluidic mixer structures that extend up from the bottom of the channel^35^,^36^. However, even with its reported increase in capture efficiency, this system had a somewhat limited bead capacity. Another remedy has been to use magnetically activated separation (MACS) columns, in which a high gradient magnetic field is induced by a packed spherical steel particle matrix when magnetized by an external magnetic field. MACS is fast and relatively efficient for batch-mode cell separation. However, clogging problems arise due to the deposition of magnetic cells on the matrix, which limits the separation capacity when applied to continuous-flow microfluidic systems.

In general, the aforementioned magnetic configurations are limited by the range of the magnetic capture force that they provide, which is typically only effective for particle capture within tens of microns of the elements. Therefore, they are only useful for relatively narrow microchannels, which limits system throughput. The proposed use of flow-invasive magnetic elements overcomes this limitation by producing a viable capture force throughout the entire flow channel. Moreover, the viability and superior capture performance of the proposed microscale system can be inferred from successful implementation of macroscale separation and filtration systems that utilize transverse flow invasive elements, which have been shown to have excellent capture efficiency. One such system with dimensions on the order of centimeters employed a 2D matrix of transverse soft-magnetic wires in a flow channel to capture micron-sized magnetic particles^37^. In another related system, a matrix of millimeter-scale flow-invasive soft-magnetic rods was used to separate suspended magnetic particles^38^. While many challenges exist in integrating flow-invasive elements in microfluidic systems, our theoretical work indicates that the proposed design merits fabrication as it provides a significant improvement in capture efficiency, scalability and throughput as compared to conventional systems.

We demonstrate enhanced separation efficiency for flow-invasive magnetic elements using a computational fluid dynamic (CFD)-based model that takes into account the dominant mechanisms of particle transport. The model is described in detail in the Methods section below. Briefly, a hybrid computational approach is taken in which particle and fluid transport are computed numerically using CFD, but the magnetic force, which causes particle capture, is obtained in closed-form. The transport analysis is based on a Lagrangian-Eulerian numerical approach that takes into account magnetic and hydrodynamic forces and coupled momentum transfer between the particles and the fluid^12^,^19^,^39^. The use of analytical magnetic analysis greatly improves the efficiency and accuracy of the magnetic force computation by eliminating computationally intensive numerical field modeling. Similar albeit less rigorous models have successively predicted capture efficiency for macrocale magnetic filtration systems with transverse flow-invasive elements^37^. We use our model to quantify for the first time the enhanced capture efficiency provided by this magnetic configuration at the microscale. The use of such flow-invasive elements opens up opportunities for the development of microfluidic systems with superior capture efficiency, scalability and throughput. The computational model enables the rational design of such systems.

## Results

**Figure 1:**
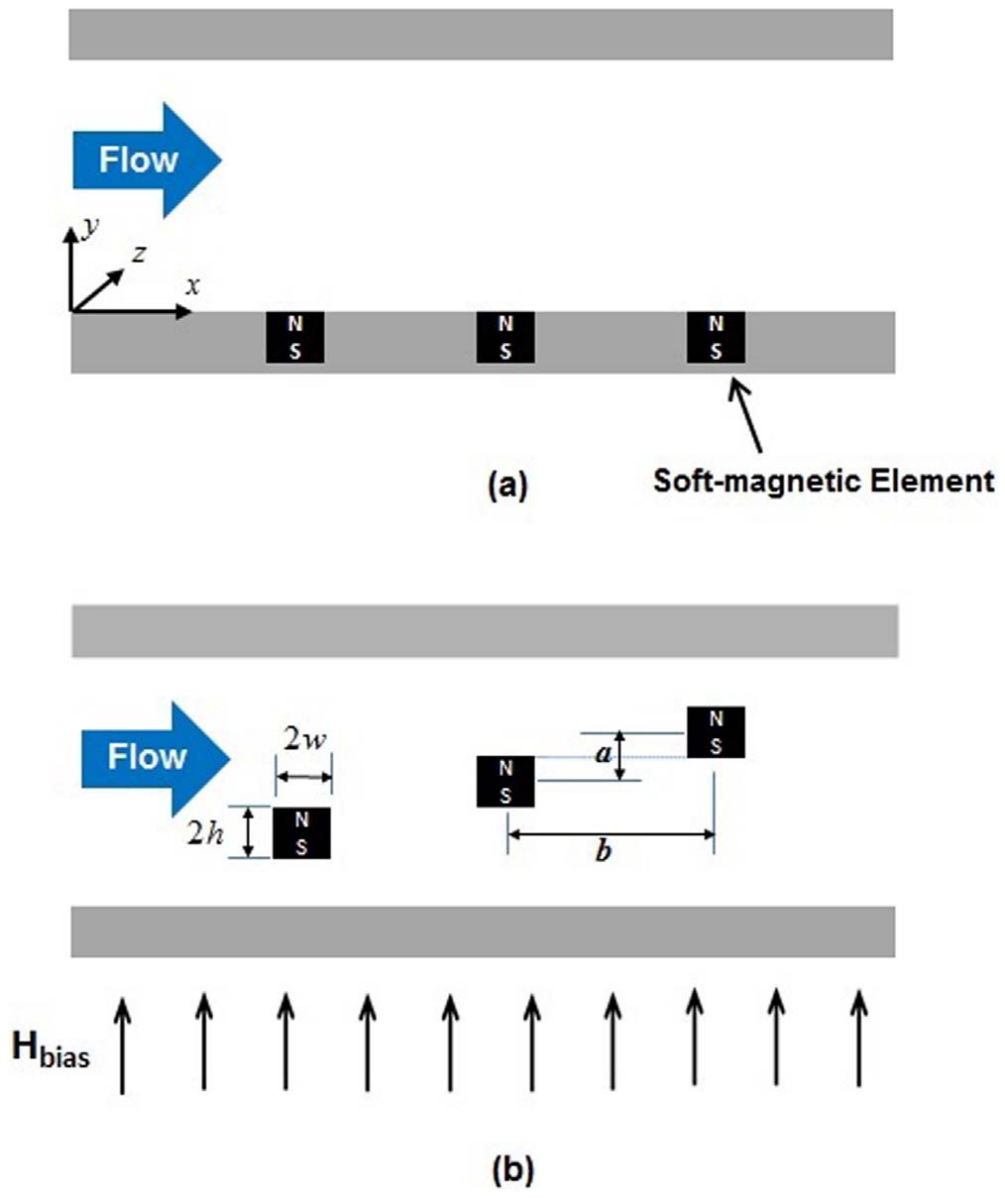
Schematic diagrams showing cross-sectional side-views of a microchannels with soft-magnetic elements (a) conventional system with elements embedded in the base of the microchannel, and (b) proposed system design with flow-invasive elements arranged in a stair step configuration.

We use the computational model to study particle separation for two distinct microfluidic systems that are shown schematically in Figure 1. The first system, illustrated in Figure 1a, represents a more conventional magnetic configuration wherein soft-magnetic elements are embedded in the base of a microchannel. The second system, shown in Figure 1b, represents the proposed configuration wherein the same elements are arranged inside the microchannel, transverse to the flow field in a vertical stair step arrangement that spans the height of the microchannel. For the purpose of analysis, the microchannel is taken to be 200 μm high (y direction) and 10 mm long (x direction). We assume that the channel width (into the page) is considerably larger than its height and that the magnetic elements extend lengthwise along the entire width of the channel. The elements are assumed to be made from permalloy (78% Ni, 22% Fe), which has a saturation magnetization *M_es_* = 8.6 × 10^5^ A/m. They have a nominal cross-sectional height and width of 40 μm. In Figure 1a the elements are spaced 80 μm apart, center-to-center. In Figure 1b, they have the same horizontal center-to-center spacing (i.e. *b* = 80 μm) but are ordered in a flow-invasive vertical stair step arrangement with a vertical center-to-center spacing of *a* = 40 μm. The magnetic particles used in the analysis are the MyOne™ beads from Dynal Biotech (www.dynabead.com), which have a radius *R_p_* = 0.5 μm, density *ρ_p_* = 1800 kg/m^3^, saturation magnetization *M_sp_* = 4.3 × 10^4^ A/m and an initial (low field) apparent susceptibility *χ_a_* = 1.4. A uniform bias field of *H_bias_* = 3.9 × 10^5^ A/m (0.5 Tesla) is applied upward, perpendicular to the base of the channel, which is sufficient to saturate both the elements and the particles^12^. Fully-developed laminar flow of an incompressible carrier fluid with the properties of water, i.e. viscosity *η* = 0.001 kg/m and density *ρ_f_* = 1000 kg/m^3^, enters the microchannel at the inlet (left) with an average velocity *u_avg_*. The outlet gauge pressure is set to zero. We study particle capture in these systems using a two-dimensional (2D) CFD analysis. It suffices to consider systems with only a few flow-invasive elements (e.g. 3) as these are sufficient to achieve 100% capture efficiency.

### One-way Particle-Fluid Coupling

In this section we study particle capture using one-way particle-fluid coupling analysis. In this analysis, the motion of the particles is impacted by the fluid flow due the effects of viscosity, but the particle motion does not alter the flow, i.e. there is no reciprocal transfer of momentum from the particles back to the fluid.

#### Conventional System

**Figure 2:**
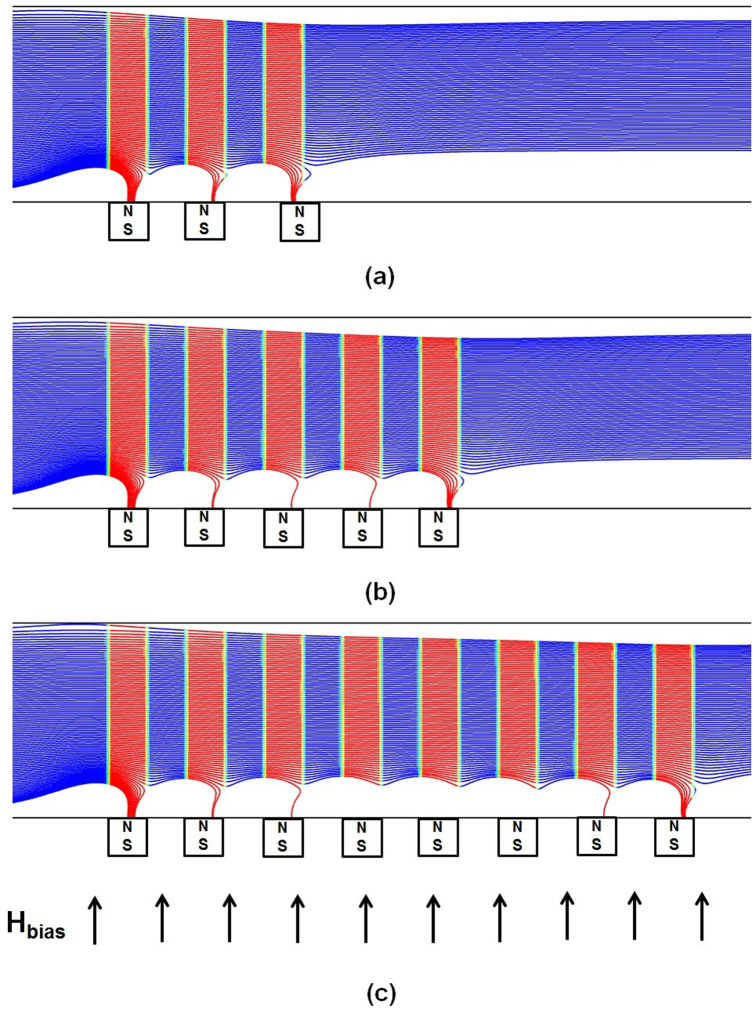
Particle trajectories for one-way particle fluid coupling in a conventional system: *u_avg_* = 1.0, 40 µm × 40 µm elements with *b* = 80 µm; (a) 3 elements, CE = 33%, (b) 5 elements, CE = 34% and (c) 8 elements, CE = 34%.

**Figure 3:**
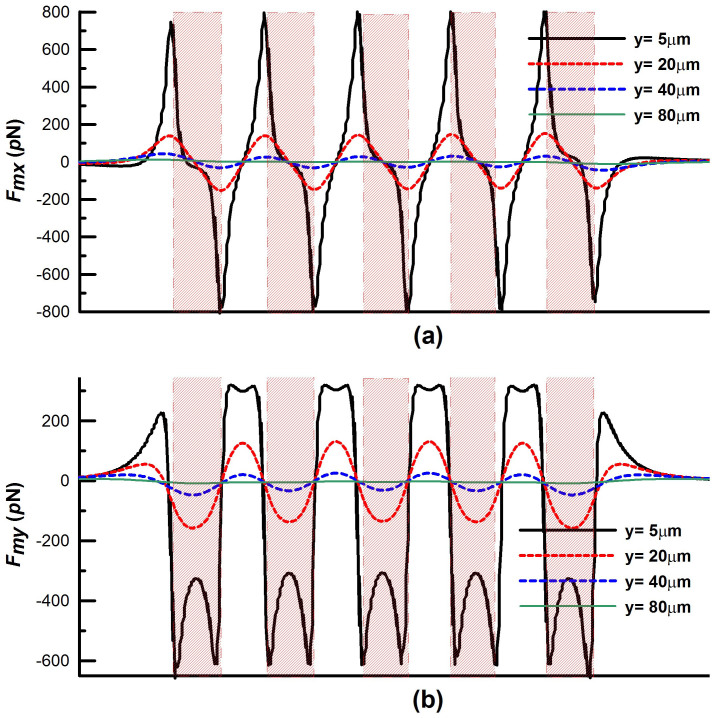
Longitudinal profiles of the magnetic force components across the microchannel above five magnetic elements: (a) *F_mx_* and (b) *F_my_* vs. the distance *y* above the elements.

We first study particle capture in a conventional bioseparation system as shown in Figure 1a. Particles in a state of dilute suspension are injected along 100 streams uniformly distributed over the inlet plane. Predicted particle trajectories along with the capture efficiency (*CE*) for this system are shown in Figure 2 for an inlet velocity *u_ave_* = 1 cm/s. With such velocity, the two dimensional (simulation) flow rate (i.e. based on a unit length in the z-direction) is 2 mL/s). For a practical three dimensional system, the physical flow rate based on, say 1000 μm depth in the z-direction, would be 2 μL/s. The particle trajectory profiles in blue are superimposed with vertical red bands that identify portions of the flow directly above the magnetic elements, i.e. the red bands span the width of the embedded elements end-to-end. Figure 2a shows that only 33% of the injected particle streams are captured using three embedded elements, mostly at the leading element and to a lesser extent at the trailing element, essentially bypassing the second element. As noted earlier, a major disadvantage of conventional embedded element systems is the short-range effective capture distance of the magnetic force, which is limited to within tens of microns of the elements. It is instructive to examine this force in detail. The horizontal and vertical magnetic force components *F_mx_* and *F_my_* at various distances above a five element system are shown in Figures 3a,b. Note that the force decays rapidly with distance. Specifically, at a distance above the elements equal to their height (H = 40 μm), the force has decreased an order of magnitude from its maximum value (i.e. from hundreds to tens of pN). Thus, the capture force greatly diminishes for particle paths outside of a threshold distance, which depends on both the dimensions of the elements and the flow velocity. As the channel dimensions increase the overall capture efficiency decreases, which limits the throughput.

The variation of the force (in the direction of flow) also impacts capture efficiency. As shown in Figure 3b the vertical force *F_my_* is upward (repulsive) just to the left and right of an element but downward (attractive) above the element. This implies that the particle will accelerate upward, away from the element, near its edges. The reason for this is that the value of the field gradient, which is due to the magnetized elements, changes its sign within the microchannel. On the other hand, the bias field imposes an upward magnetization of the particle throughout its travel. Since the force is a product of the induced magnetic moment times the gradient (see Eq. (2)), it is positive (repulsive) when both terms are positive and negative (attractive) when the terms have opposite signs. The alternating polarity of *F_my_* produces an oscillatory up-and-down motion in the particles as it passes an element. The presence of an upward force, which is strongest above the gaps between the elements, reduces the overall capture efficiency as it pushes the particles away from the elements.

**Figure 4:**
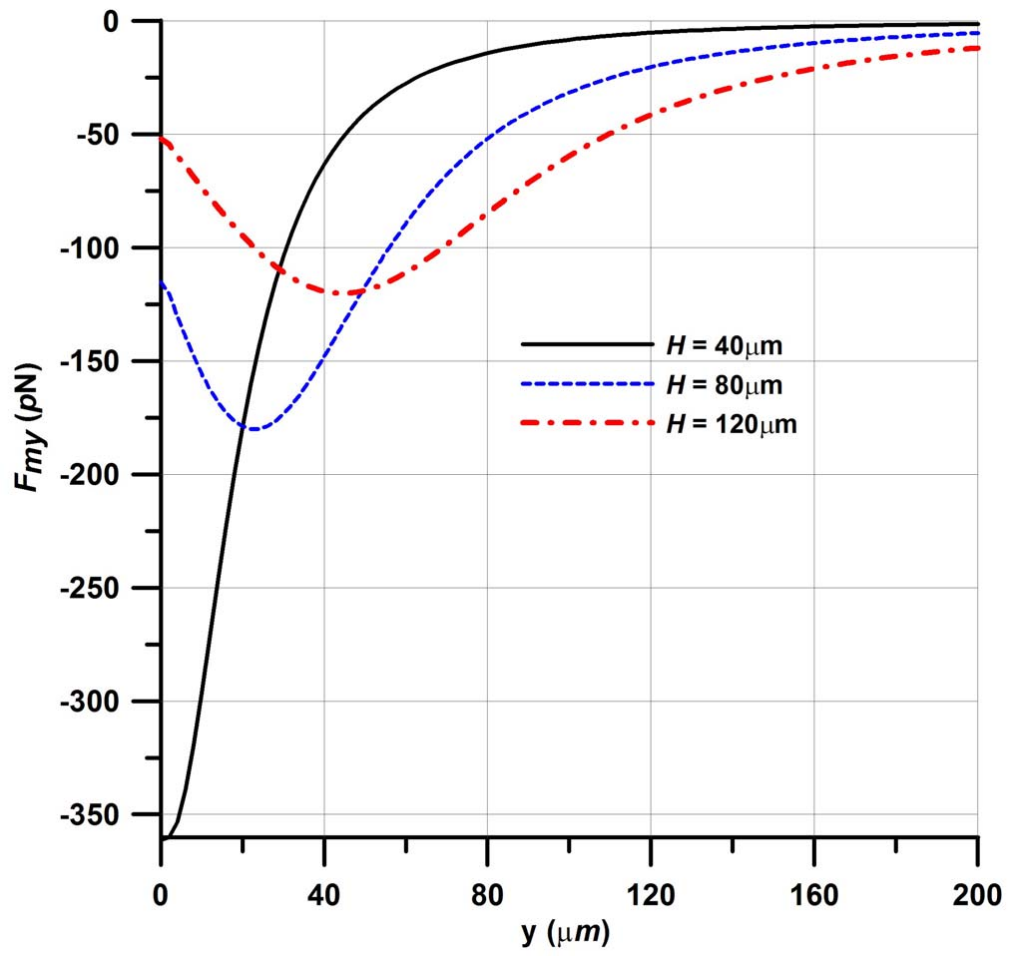
Magnetic capture force *F_my_* above a square element of side H along a vertical centerline from the top of the element (y = 0) to the top of the microchannel (y = 200 μm).

Next, we investigate how the capture force scales with the size of the elements. Recall that the nominal magnetic elements are square, with dimensions H = W = 40 μm. We compute the capture force *F_my_* above the center of a single element along a vertical line that runs from its top surface (y = 0) to the top of the microchannel at y = 200 μm. The force profiles for three different sized square elements, 40, 80 and 120 μm on a side, are plotted in Figure 4. Notice that the peak force near the top of the element decreases dramatically from 361 pN to 115 pN to 52 pN as the element increases from 40 μm to 80 μm to 120 μm, respectively. This is because the larger elements produce a reduced field gradient. This analysis shows that in general the capture force decreases rapidly with distance from the elements and that larger elements produce a weaker capture force near their surface than smaller elements.

Finally, we show that the capture efficiency exhibits poor scalability, i.e. there is little improvement in particle capture as the number of elements increase. This is evident in Figures 2 (a-b-c), which show that there is no tangible increase in capture efficiency when the number of elements increases from 3 to 5 to 8, respectively.

#### System with Flow-Invasive Elements

**Figure 5:**
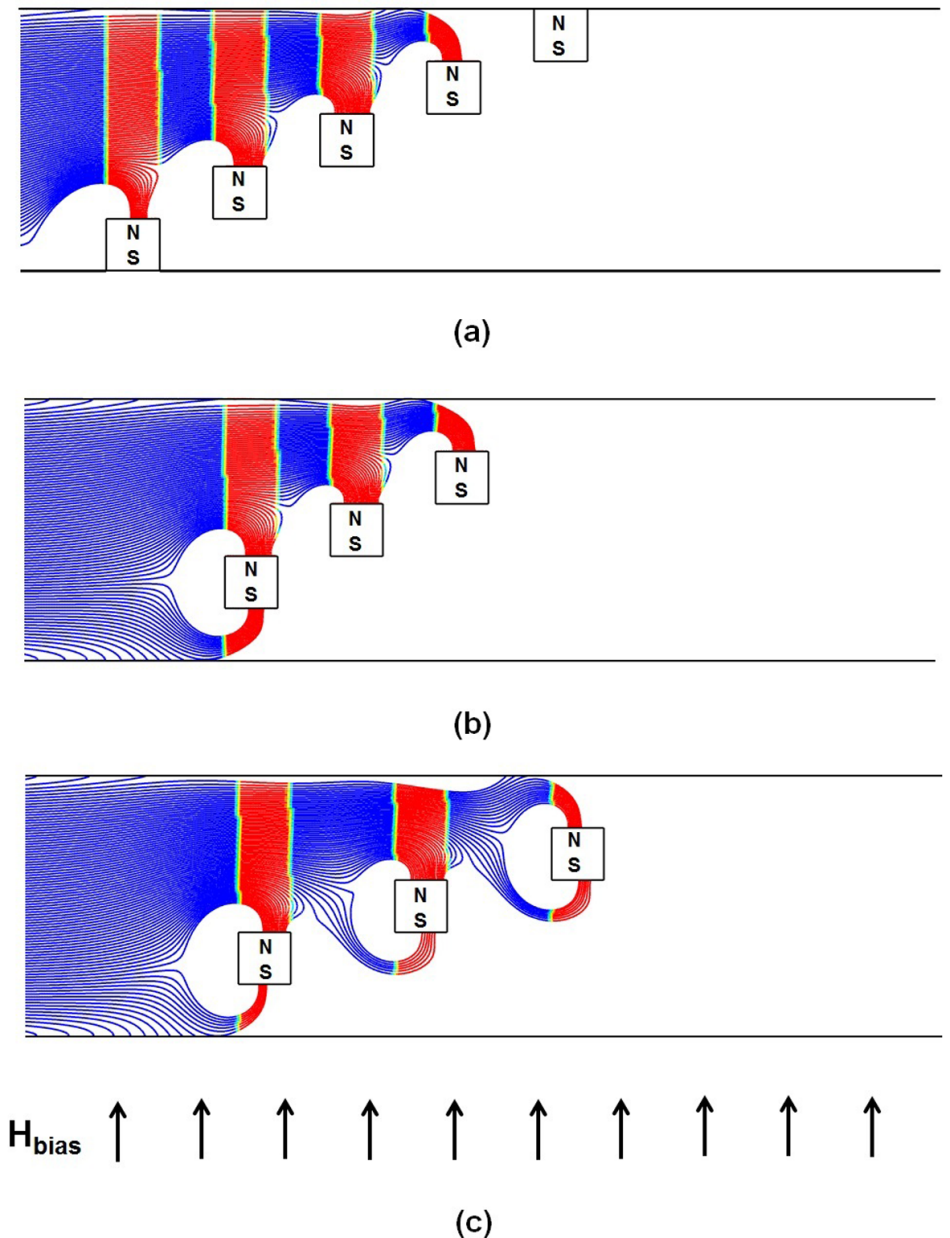
Particle trajectories for one-way particle fluid coupling in the proposed system: *u_avg_* = 1.0 cm/s, 40 µm × 40 µm elements with *a* = 40 µm; (a) 5 elements, *b* = 80 µm, CE = 100% (b) 3 elements, *b* = 80 µm, CE = 100% and (c) 3 elements, *b* = 120 µm, CE = 100%.

**Figure 6:**
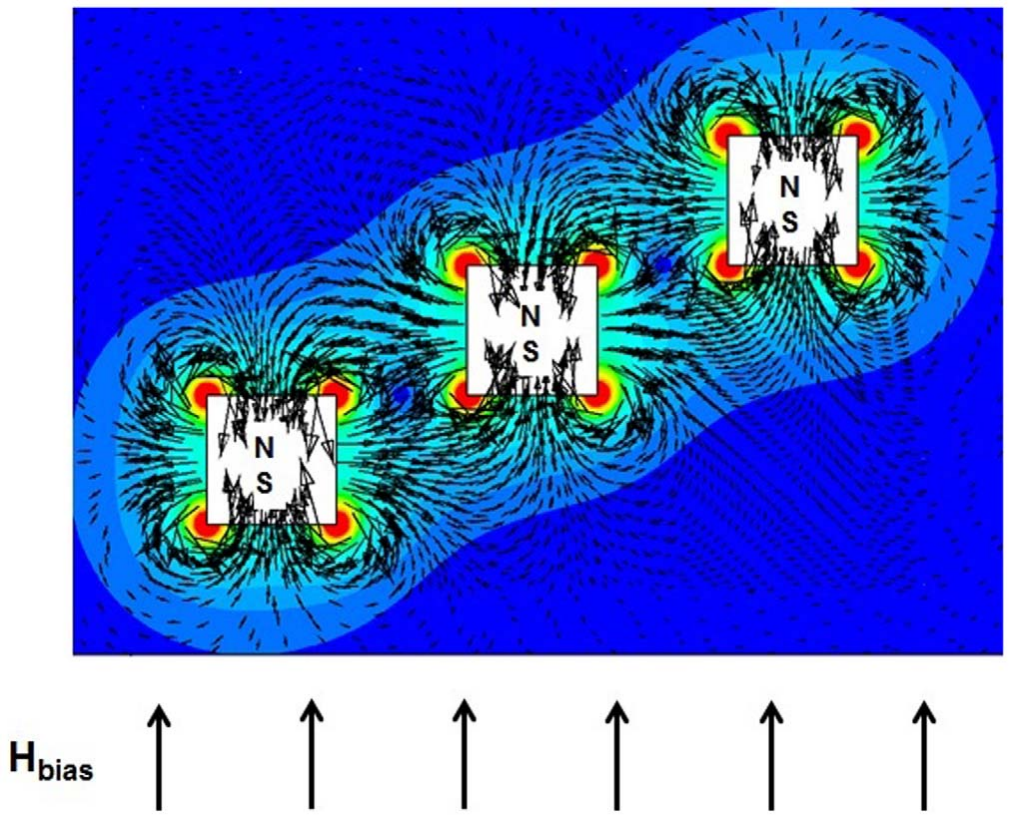
Magnified view of the magnetic force field due to three 40 µm × 40 µm flow-invasive elements overlaid by the contours of the force magnitude (*a* = 40 µm, *b = * 80 µm).

We now consider particle capture for the proposed design with flow-invasive elements. Particle trajectories for this configuration are shown in Figure 5. Here, the short-range magnetic force is now cascaded-up in a manner that enables efficient particle capture throughout the entire channel height. This produces a greatly improved capture efficiency of *CE* = 100%. Such value indicates that none of the particles escaped the downstream end of the microchannel. Less than 5% of the particles are observed to indefinitely trapped at the upper and, to a much less extent, at the lower walls due to the repulsive force described earlier. In that sense, the capture efficiency CE adopted here reflects the filtration efficiency of the system. An important feature of this configuration is that the magnetic force is attractive on the top and bottom surfaces of an element and repulsive on its vertical sides. This is due to the superposition of the uniform bias field and the localized gradient-fields of the elements as described above. This behavior is illustrated in Figure 6, which is a magnified view of a plot of the magnetic force vectors for a three element array, overlaid by contours of the force magnitude 
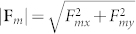
. Note that both the attractive (vertical) and repulsive (horizontal) force vectors increase dramatically near the corners of the elements. This is because the field gradient changes sign abruptly in these regions. This gives rise to disproportionately large field vectors at the corners, which have been suppressed in the plot in order to clearly display the force vectors on the surfaces.

Referring again to Figure 5, note that particle capture takes place exclusively on the top and bottom surfaces of the elements where the force is attractive. No particles accumulate on the vertical sides. Significantly, the repulsive force on these sides helps to focus the particles towards the top and bottom surfaces of adjacent downstream elements to facilitate capture. In fact, for the 5 element arrangement shown in Figure 5a in which the first element is positioned on the base of the channel, the particle capture takes place exclusively on the top sides of the elements due to aforementioned focusing. This focusing decreases as we increase the axial spacing between elements from 80 to 120 µm as shown in Figure 5c.

### Two-way Particle-fluid Coupling

**Figure 7:**
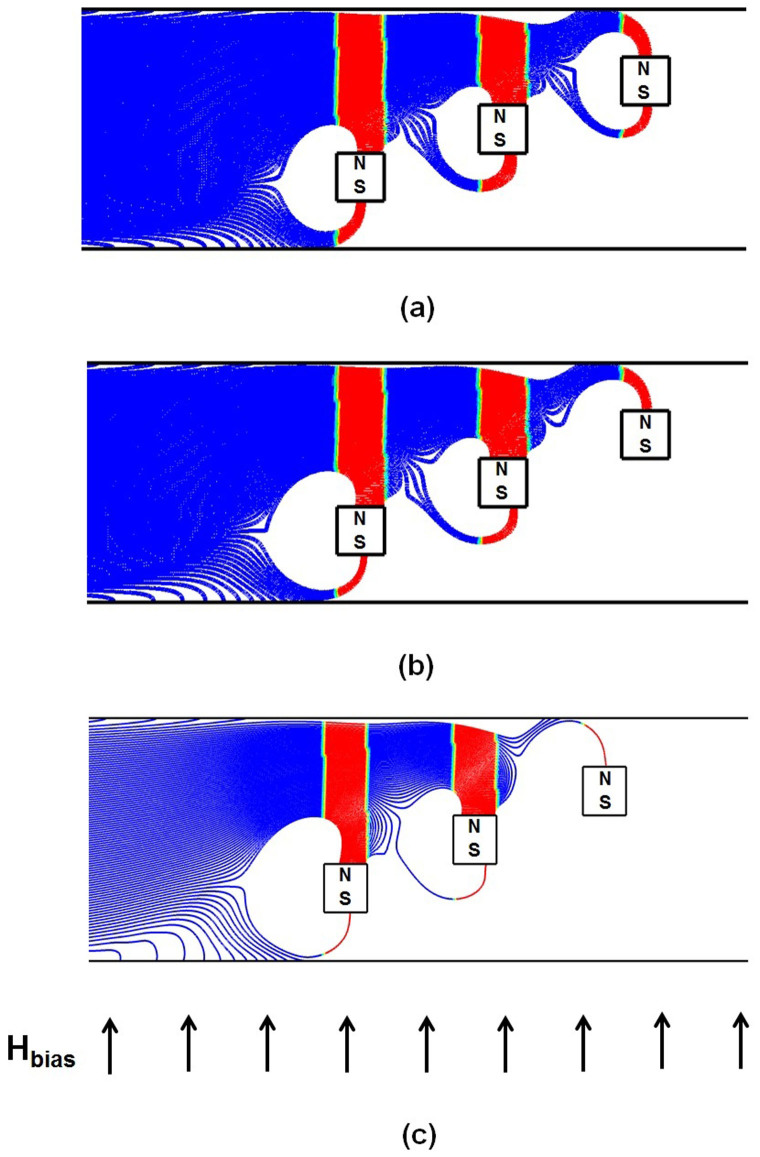
Particle trajectories for two-way particle-fluid coupling (*u_avg_* = 1.0 cm/sec, 40 µm × 40 µm elements with *a* = 40 µm and *b* = 120 µm) for inlet particle-to-fluid volume ratios of; (a) 0.028%, (b) 0.084% and (c) 0.14%.

**Figure 8:**
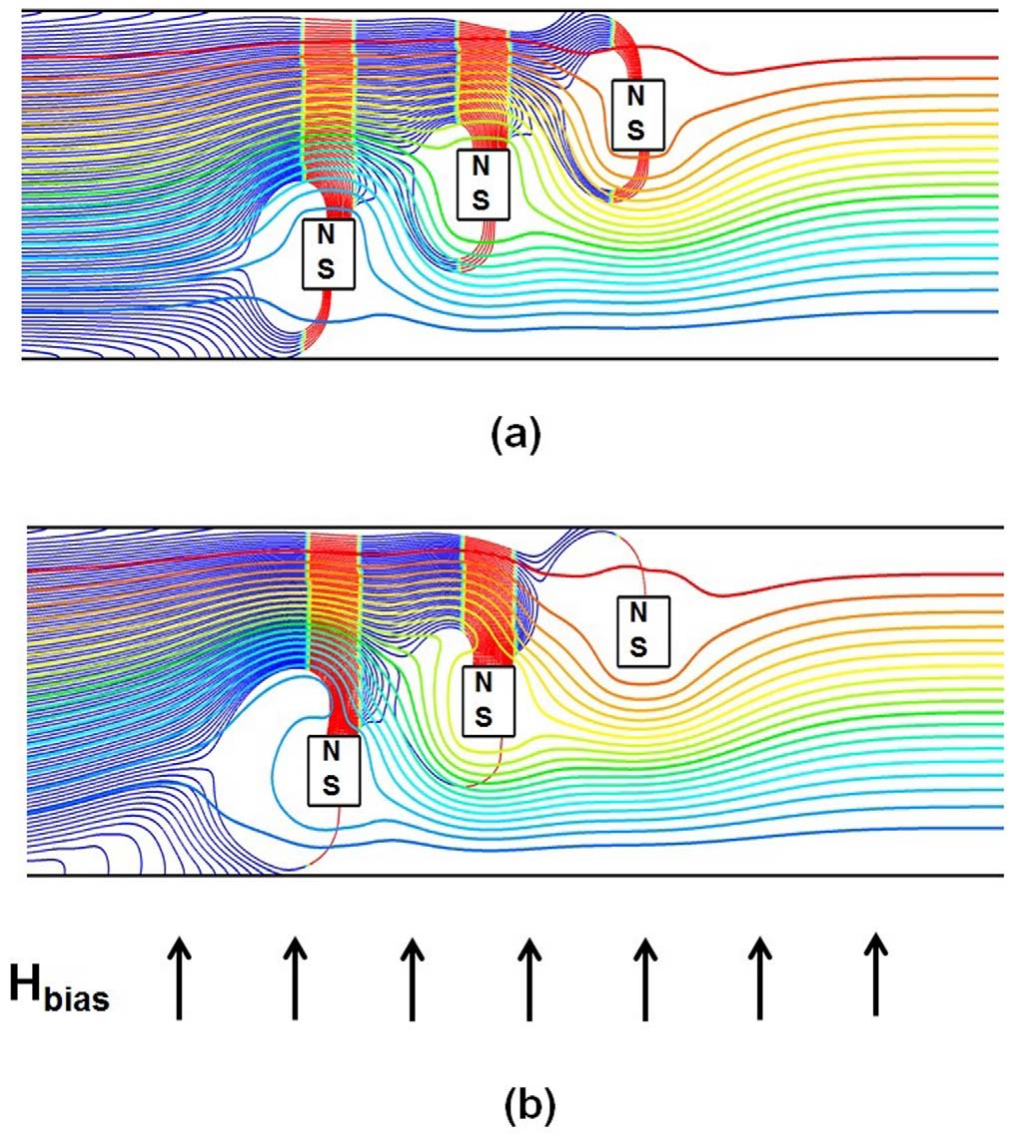
Predicted particle trajectories and the flow stream function *ψ* for the highest injected particle-to-fluid volume ratio of 0.14% and with *u_avg_* = 1.0 cm/sec (*a* = 40 µm, *b = * 120 µm): (a) one-way coupling and (b) two-way coupling and. (two-dimensional *ψ_min_* = 1 × 10^−4^ kg/s, *ψ_max_* = 2 × 10^−3^ kg/s).

In this section we investigate the capture efficiency for flow-invasive elements as a function of the injected particle loading. This analysis takes into account fully-coupled particle-fluid interactions wherein the motion of the particles is impacted by the fluid flow and the particle motion, in turn, alters the flow, i.e. there is a transfer of momentum from the particles to the fluid. The analysis is performed using three 40 µm × 40 µm elements with *a* = 40 µm and *b* = 120 µm. The predicted particle trajectories for three different particle volume fractions of 0.028%, 0.084% and 0.14%, are shown in Figure 7. The corresponding particle-to-fluid mass ratios are 0.05%, 0.15% and 0.25%, respectively. For the lowest volume fraction the calculated trajectories are almost identical to those obtained using one-way coupling, i.e. Figure 5c vs. Figure 7a. However, the effects of two-way coupling becomes significant as the injected volume ratio increases to its highest level (Figure 7c). This level of loading produces higher capture efficiency because of a cooperative effect between the particle motion and flow. Specifically, as the particles accelerate towards the top or bottom surfaces of the elements due to the attractive magnetic force, they “pull” the flow towards these surfaces as well due to a reciprocal Stokes' drag, which in turn promotes enhanced particle capture as shown in Figure. 8. It is worth mentioning that the streamlines presented for the one-way coupling in Figure 8a, were found to be almost identical to those present under two-way coupling in the absence of magnetic field. The model also predicts that even when the particle loading is dilute at the injection plane, the local volume fraction can be substantially higher due to two-way particle-fluid coupling near capture regions where the particles congregate. In fact, using a transient simulation (not shown here) we find that the local particle volume fraction can be several times greater than the initial injected loading due to particle-fluid coupling, which could lead to particle aggregation. As the main focus here is to provide a proof-of-concept, we did not attempt to conduct a rigorous optimization study for the new design. However, numerical simulations under different inlet velocities, particularly to the 3-elments configuration (b = 120 µm), revealed that the 100% particle retention (>95% at the invasive elements exclusively) can be attained up to an inlet velocity of 1.25 cm/s. The particle retention decreases to 85% and 75% at 2 cm/s and 3 cm/s, respectively.

## Discussion

We have used a computational model to demonstrate proof-of-concept of a method for greatly enhancing magnetic biosepartion in microfluidic systems using flow-invasive soft-magnetic elements. The elements intersect the carrier fluid and are arranged transverse to the flow in a vertical stair step configuration that spans the height of the flow channel. The analysis shows that the proposed magnetic capture configuration provides substantially enhanced capture efficiency relative to conventional systems where the magnetic elements are embedded in the walls of the flow chamber and do not intersect the flow. The model, which is described in more detail the Methods section, combines an Eulerian-Lagrangian CFD analysis with closed-form magnetic field analysis and can be used to predict magnetic separation taking into account dominant magnetic and hydrodynamic forces as well as two-way particle-fluid coupling. The analysis quantifies for the first time the enhanced capture efficiency of flow-invasive elements at the microscale. There are two unique features of these elements that contribute to their improved capture performance. First, the relatively short-range capture force of the elements is cascaded-up in a manner that enables efficient particle capture throughout the entire channel height. This is in sharp contrast to conventional systems wherein the elements are integrated into the walls of the flow channel. This limits the size of the flow channel and hence throughput as the effective range of capture is typically only tens of microns from the elements. The second and less obvious feature is that the elements produce regions of attractive and repulsive magnetic force on their horizontal (top and bottom) and vertical (side) surfaces, respectively. This somewhat unique force field, which exists around each individual element, is a result of the combination of the applied bias field and the localized gradient-fields due to the magnetic elements. We have found that the regions of repulsive force tend to focus the particles towards the attractive surfaces of neighboring downstream elements thereby further enhancing capture efficiency. The model also elucidates the effects of particle-fluid coupling. When two-way coupling is considered, we find that there is a cooperative effect between particle and fluid transport that enhances particle capture but also produces localized regions of relatively high particle loading, which could lead to undesired particle aggregation. The enhanced capture is due to a particle-induced perturbation of the flow field that acts downward toward the attractive surfaces of the elements.

Overall, the modeling demonstrates that flow-invasive magnetic elements provide substantial performance advantages over conventional bioseparation systems. However, they are more challenging to integrate at the microscale. One potential method for fabrication would be a layer-by-layer approach wherein the magnetic elements are formed sequentially in situ via a sequence of spin coating, patterning, etching, deposition and planarization followed by the formation of the flow channel via removal of sacrificial material. Some challenges with this approach would be the time required for depositing the soft-magnetic elements, the precision in their patterning, the quality of their magnetic properties, their structural integrity under flow, the potential for detrimental residual stress in the system, especially in relatively long and thin elements, due to a mismatch in material properties and the ability to etch a flow channel with uniform cross-sectional dimensions with the elements in place. An alternative method would be to fabricate an array of the magnetic elements on a separate substrate and then release and articulate them into position into etched slots is microfluidic structure followed by backfilling, planarization and formation of the channel. In addition to some of the aforementioned issues, a key challenge with this approach would be the precise positioning and alignment of the submicron elements in the pre-etched slots. Variations in element and slot size and local stiction forces could make this difficult. On the other hand, for larger millimeter-scale fluidic systems, one could potentially use 3D printing to form the system and insert relatively larger magnetic elements during fabrication. In any event, the integration of flow-invasive elements in a microfluidic platform opens up opportunities for the development of separation systems with superior efficiency, scalability and throughput and as compared to conventional systems. The computational model enables the rational design of such systems in advance of fabrication and should be of considerable interest to researchers involved in the development of lab-on-a-chip systems.

## Method

We predict particle transport and capture efficiency for the proposed system using a computational model that takes into account dominant magnetic and hydrodynamic forces as well as coupled particle-fluid interactions. Our model is based on a coupled Lagrangian-Eulerian CFD-based numerical scheme combined with analytical magnetic analysis for the magnetic force. The Lagrangian analysis is used to track the motion of individual particles while an Eulerian-based CFD analysis is used to solve the Navier Stokes momentum equations. The transfer of momentum from the particles to the fluid is accomplished by introducing a particle force sink into the Navier Stokes equations. The ability to evaluate two-way particle-fluid coupling is an important feature of our model that distinguishes it from most other models used in this field that are limited to one-way particle-fluid coupling, which neglects the effects of particle motion on fluid flow. Another important aspect of our model is that we use a hybrid closed-form/numerical approach that combines the numerical transport analysis with closed-form field analysis. The use of closed-form magnetic analysis provides an exact prediction of the magnetic field and force in the microchannel and is computationally more efficient and accurate than numerical field analysis, which is more commonly used for this analysis. To this point, it should be noted that while the transport equations are solved in a confined computational domain (i.e. the microchannel); the magnetic analysis requires a larger (open) domain because the field extends to infinity. Moreover, accurate magnetic force values depend on the gradient of the field, which depends on the resolution of the grid. Typically, a large number of computational nodes are required to achieve an accurate magnetic analysis, which increases computation time and inhibits large-scale parametric analysis. The closed-form magnetic analysis used here overcomes these limitations and enables rapid parametric studies of capture efficiency for systems with extended and complex magnetic structures.

Since we use the model for proof-of-concept, it should be noted that it is based on well-established and experimentally validated theory that has been widely used for several decades to predict the transport and capture of magnetic particles in the presence of magnetized elements. Several groups have validated a less rigorous analytical Lagrangian approach for predicting particle capture of paramagnetic particle slurries in axial and transverse stream type HGMS filters that employ flow-invasive soft-magnetic (e.g. Ni) wires similar to that proposed in our system. The validation experiments were originally developed for a single wire aligned parallel to the flow by Friedlaender et al. (1978)^40^ and subsequently refined and applied for a matrix of wires by Schewe et al.(1980)^41^; Takayasu et al.(1983)^42^; Gerber et al.(1984)^43^ as well as many others over the last 30 years. As part of this progress, experimental particle trajectories have been compared to predicted trajectories and found to be in good agreement^41^. In addition, macroscale magnetic filtration systems that employ transverse flow-immersed soft-magnetic wires, very similar to the configuration proposed here, have been fabricated and analyzed using modeling approaches similar to ours but less accurate due to the use of analytical solutions or reduced Navier Stokes equations to predict the flow field. Significantly, even these less rigorous models have provided good agreement with measured capture performance^44^.

We now describe our model in more detail. As noted above, we use a Lagrangian formulation to predict particle notion. The transport of colloidal magnetic particles under the influence of an applied magnetic field is a complex function of several factors, among these are magnetic and hydrodynamic forces including interparticle effects, Brownian motion and Van Der Waal's force, etc. For the particle size and concentration considered in this study, all effects can be neglected except for the dominant magnetic and hydrodynamic forces. We predict particle transport using Newtonian dynamics^12^,^17^,^19^, 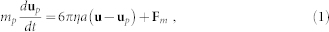
where **u** and *η* are respectively, the velocity and viscosity of the carrier fluid and *m_p_*, **u_p_**, *a* and *V_p_* are, respectively, the mass, velocity, radius and volume of the particle. The first term on the right hand side represents the fluidic drag as defined by Stokes' law and the second term is the magnetic force. The latter is predicted using an effective dipole moment approach in which the particle is treated as an equivalent point with a magnetic dipole moment ***m****_eff_*. The magnetic force is given by 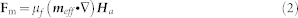
where *μ_f_* is the permeability of the carrier fluid and **H**_a_ is the applied magnetic field intensity (A/m) at the center of the particle. The magnetic dipole moment is given by *m_eff_* = *V_p_M_p_*where *V_p_* and *M_p_* are the volume and magnetization of the particle, respectively. It can be determined using a magnetization model that takes into account self-demagnetization and magnetic saturation, 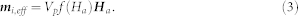


In this model the magnetization is assumed to be a linear function of *H*_a_ up to saturation, after which it remains at the saturation value *M_sp_*. The function *f*(*H*_a_) can be written as^12^,^17^,^39^: 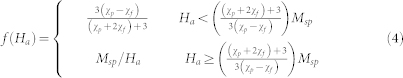
where *χ_f_* is the susceptibility of the fluid and *χ_p_* is the intrinsic magnetic susceptibility of the particle, i.e. *M_p_* = *χ_p_H_in_* where *H_in_* is the field inside the particle. *H_in_* differs from *H_a_*by the demagnetization field i.e. *H_in_* = *H_a_*−*N_d_M* where *N_d_* is the demagnetization factor of the particle, i.e. *N_d_ = * 1/3 for a spherical particle. The value of *χ_p_* can be obtained from a measured *M* vs. *H* curve. However, *M* is often plotted as a function of the applied field *H_a_* in which case *M_p_* = *χ_a_H_a_* where *χ_a_* is the apparent susceptibility. The two values of susceptibility are related as follows *χ_p_* = *χ_a_*/(1−*N_d_χ_a_*), which reduces to *χ_p_* = 3*χ_a_*/(3−*χ_a_*) for a spherical particle^27^. In our analysis we assume that the carrier fluid is nonmagnetic i.e. *χ_f_* = 0. Taking the above into account, equation (1) can be rewritten as 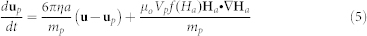
The trajectory of a particle is defined using the kinematic equation, 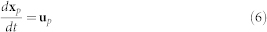
Equations (5) and (6) can be integrated using a variety of numerical techniques such as the fourth-order Runge-Kutta method, which we use in our analysis.

An expression for the magnetic field **H*_a_*** is needed for the solution of equation (5). We use a closed-form analytical expression, which is briefly presented here for convenience. A detailed derivation can be found in the literature^12^,^17^,^45^. The components of the magnetic field **H**_e_ for a long rectangular element of width W = 2*w* and height H = 2*h* that is centered with respect to the origin in the x-y plane, and magnetized to saturation *M_es_* by a bias field *H_bias_* in the vertical direction (along the y-axis) can be expressed as 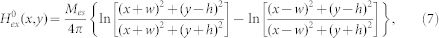
and 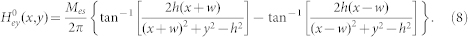
The magnetic force components for a single element are 
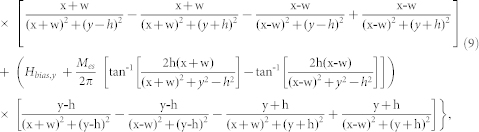
and 
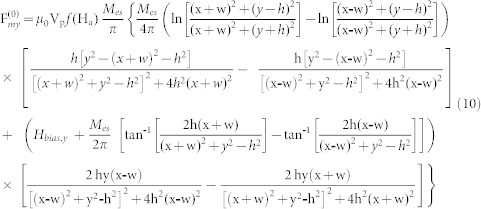
The field and force for an array of elements can be obtained from equations (7) and (8) using the principle of superposition. Specifically, let *N_e_* denote the number of elements in the array, and let *n* = (0, 1, 2, 3, 4,…, *N_e_*-1) label the individual elements. Equations (7) and (8) denote the field components due to the first element (*n* = 0). The *n*'*th* element is centered at (x_n_, y_n_) and its field components can be written as 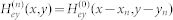 and 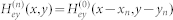. The total field of the array is obtained by summing the contributions from all the elements, 
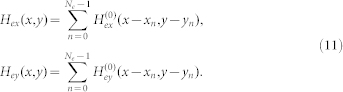
It follows that the force components are 
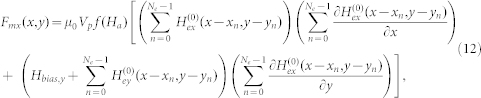
and 
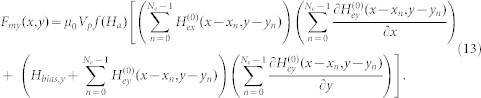
In equations (12) and (13), we have assumed that the bias field is uniform and in the y-direction. Additional useful models for magnetic structures can be found in the literature^46^,^47^,^48^,^49^.

The flow-invasive elements give rise to a complex velocity field **u** within the flow channel. This is determined numerically by solving Navier Stokes equation under the assumption of incompressible flow ∇·**u** = 0, 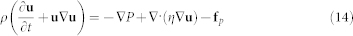
Here *P* is the fluid pressure and **f**_p_ is a volumetric (particle-weighted) sink term that accounts for the counter drag force impacted to the fluid by the particles, i.e. two-way particle-fluid coupling. Coupled particle-fluid transport is predicted using finite-volume based computational fluid dynamics (CFD) analysis. The two-way coupling is implemented using the discrete phase model (DPM) available in ANSYS FLUENT (www.ANSYS.com). The DPM employs a combined Euler-Lagrange approach in which the Navier-Stokes equations are solved using a continuum Eulerian framework, while the trajectories of the dispersed phase are solved for representative particles or parcels using a discrete Lagrangian formulation^12^,^19^,^39^. In the standard DPM, each parcel represents a collection of particles and the behavior of each parcel is determined by the behavior of its constituent particles. If the coupling is one-way, i.e. fluid impacts particles but not vice versa, the difference between single particle and a parcel is immaterial. However, the concept of parcel is relevant for two-way coupling since the counter drag sink term **f**_p_ is calculated based on the particle density (concentration) present in each computational cell. In our study, a parcel is subjected to a magnetic force and fluidic drag. A closed-form expressions for the magnetic field and the generated magnetic force are calculated, independently from the fluidic field using a User Defined Function (UDF). The particle-fluid momentum transfer is^12^,^19^,^39^

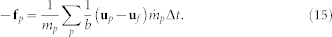
In equation (15) the variables 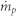 and Δt are the mass flow rate of a parcel and its time spent while crossing a continuum computational cell.

Finally, we briefly discuss the size of the microchannel that we used for our proof-of-concept simulation. We chose a channel that is 200 microns in height with consideration for a large and growing number of bioseparation applications that utilize commercial magnetic beads that range in size from 0.5 to 10 s of microns in diameter. In order to avoid undesired aggregation of the beads during flow through, the channel should be much larger than the bead diameter (i.e. more than ten times), which places the minimum height of the channel at 100 microns. Similarly, for applications that involve the separation of magnetically labeled cells, the flow channel should be much larger than a single cell. For example, circulating tumor cells (CTSs) range in size from 12–25 microns and the standard procedure to count CTCs involves a 7.5 ml blood sample^50^,^51^. If the sample needs to be diluted, then larger sample volumes are needed to be processed, which requires larger channels. Smaller channels (~100 µm) would result in the clogging of cells and an increase in their residence time before separation. However, increasing the size of the channel and the flow rate is problematic using conventional magnetic separation configurations for the reasons described above. With the proposed method, increasing the size of the separation channel does not affect the separation efficiency because of the uniform distribution of the magnetic force throughout the channel.

## Author Contributions

S.K. designed the project and conducted the CFD modeling and simulations. S.K. and E.F. analyzed the results. S.K., A.A. and E.F. wrote the paper.
